# Micro-mechanical performance evaluation of expansive soil biotreated with indigenous bacteria using MICP method

**DOI:** 10.1038/s41598-021-89687-2

**Published:** 2021-05-14

**Authors:** Nitin Tiwari, Neelima Satyam, Meghna Sharma

**Affiliations:** Department of Civil Engineering, Indian Institute of Technology Indore, Indore, India

**Keywords:** Engineering, Civil engineering

## Abstract

This study explored the effect of indigenous bacteria present in the soil to stabilized swelling behavior and improving the mechanical property of expansive soil. The objective of the research is to investigate the effectiveness of the biostimulation microbial induced calcite precipitation (MICP) for controlling the swelling-shrinkage behavior and improving shear strength of expansive soil. An attempt was made to develop an effective procedure to culture the indigenous bacteria for treating clays with varying plasticity and improve their engineering behavior. The detailed procedure has been investigated to effectively apply the MICP technique in clay soil, considering its low permeable nature. The applicability of biostimulation to clayey soils in minimizing their swelling potential and improving the strength is assessed. Both macroscale and microscale studies were conducted on untreated and biostimulated soils to observe changes in plasticity, strength, swelling, mineralogical, chemical characteristics. The present method has shown an effective alternative to improve the road pavement subgrade without affecting the eco-system of natural soil. The method investigated the effective way of providing the enrichment and cementation solution in clayey soil, which is the major concern in current literature. The study confirms that the calcite content has been increased with biostimulated MICP treatment up to 205% in the treated specimens and which future increased the unconfined compressive strength and split tensile strength. A reduction in the swelling pressure and swell strain is also observed. The results show that a cost-effect and eco-friendly method can be deployed for stabilizing the road pavement subgrades. The statistical assessment using multivariate analysis and hierarchical clustering dendrogram has been carried out to investigate the effect of the MICP treatment protocol on different soil and engineering parameters.

## Introduction

The presence of hydrophobic minerals in the expansive soil sternly impacts its performance to use as a foundation material for civil engineering construction. The clay minerals, i.e., kaolinite, montmorillonite, and illite induce higher swelling-shrinkage nature in the expansive soil due to variation in moisture content. The expansive soil, also termed as problematic soil, covered almost one-fifth of the total area of India and the United States and can be found in several other countries^1–3^. The presence of expansive soil causes severe damage to existing structures and increases maintenance costs. The expansive soil due to the hydrophobic clay mineral presence induced higher upward swell pressure and therefore adversely affect the lightweight structures constructed over it. The various damages such as reflective cracks, lifting of structure, damage to basement, uneven settlement and cracks in walls of ceilings of the building^4,5^. Therefore, geotechnical engineers and practitioners are striving hard to develop sustainable ground improvement techniques to mitigate the adverse effect of expansive soil^6–8^. Damages caused by expansive soil can be observed around the world; approximately $ 7–9 billion per year economic losses have been reported in the United States alone^9^.

Several mechanical, chemical, and mecho-chemical methods have been developed to improve the expansive soil engineering property with varying success rates^10–15^. Various industrial waste and traditional materials such as lime, bottom ash, fly ash, pond ash, biochar, silica fume cement are majorly used to stabilize the expansive soil^16–20^. The various mechanical stabilization, i.e., geosynthetic installation, fiber reinforcement, EPS reinforcement, compaction, are also used as viable stabilization methods^21–25^. Although these techniques have shown a high improvement in the expansive soil, these chemical stabilizers also adversely affect the environment^26^. In addition, these chemical stabilization techniques alter the chemical properties of the soil; as a result the contamination, toxic and hazardous characteristics, and high pH values have been observed in surrounding soil and water bodies due to leaching of these materials^27–29^. Therefore, the alternative for ground improvement technique with environment-friendly, less invasive, and durable materials are in need of time, which can control the plasticity nature of expansive soil.

Recently, the multidisciplinary, nature's biology-based microbial induced calcite precipitation (MICP) method has emerged as an eco-friendly, sustainable ground improvement technique. MICP is an innovative and promising technique for ground improvement which uses the bacterial phase of soil to precipitate Calcite in-situ via ureolysis. The ureolysis process includes hydrolysis of urea in the presence of urease enzyme, secreted through indigenous urease producing bacteria present in the soil. The urease enzyme act as a catalyst that decomposes the urea to produce one mole of ammonia and carbamate (Eq. 1). Spontaneously, the carbamate decomposes to generate ammonia and carbonic acid (Eq. 2). Two ammonia moles hydrolyzed into ammonium and hydroxide ions, which simultaneously increases the pH (Eq. 3). The carbonic acid equilibrates in bicarbonate, which further reacts with hydroxide ions and produces carbonate ions (Eqs. 4, 5). Calcium source generates Ca^2+^ ions to the soil system and resulted in calcite precipitation after reacting with carbonate ions (Eq. 6). The biogeochemical reactions associated with calcite precipitation are modified from Burne and Chen^30^.

1 $${\text{CO(NH}}_{{2}} {)}_{{2}} {\text{ + H}}_{{2}} {\text{O }}\xrightarrow {{\text{Urease enzyme}}} {\text{ NH}}_{{3}} {\text{ + NH}}_{{2}} {\text{COOH,}}$$

2 $${\text{NH}}_{{2}} {\text{COOH + }} {\text{H}}_{{2}} {\text{O }} \to {\text{ NH}}_{{3}} { + } {\text{H}}_{{2}} {\text{CO}}_{{3}} ,$$

3 $${\text{2NH}}_{{3}} {\text{ + 2H}}_{{2}} {\text{O }} \to {\text{ 2NH}}_{{4}}^{ + } {\text{ + 2OH}}^{ - } ,$$

4 $${\text{H}}_{{2}} {\text{CO}}_{{3 }} \leftrightarrow {\text{ H}}^{ + } {\text{ + HCO}}_{{3}}^{ - } ,$$

5 $${\text{HCO}}_{{3}}^{ - } {\text{ + 2OH}}^{ - } { } \leftrightarrow {\text{ CO}}_{{3}}^{2 - } {\text{ + H}}_{{2}} {\text{O}} ,$$

6 $${\text{Ca}}^{{2 + }} {\text{ + CO}}_{{3}}^{2 - } { } \to {\text{ CaCO}}_{{3}} \downarrow .$$

MICP, also known as biomineralization or biocementation because it allows the formation calcite crystals and generate cementation between soil grains. The biocementation method was found potentially significant for scaling down the carbon emissions. Based on the bacterial treatment for MICP, it is classified in two categories i.e., bioaugmentation and biostimulation^31^. The exogenous ureolytic bacteria are being added in the soil for treatment in the process of bioaugmentation. The use of native bacteria for biocementation is known as biostimulation method of MICP^31^. An extensive study on bioaugmentation treatment of sandy and silty soil was carried out in the past^32–34^. Despite an extremely detailed understanding of MICP and effective filed trial, infection and cultivation of the bacterial strains hinder the effectiveness of the method to be considered as a cost-effective alternative. Even considering the environmental aspect, the addition of the bacterial strain can unbalance the natural eco-system (presence of native bacteria), survivability of exogenous bacteria, uneven distribution, longer time needed for the permeation of bacteria, costly for the cultivation, and special cautions required when mixing. Therefore, it poses huge challenges to implement the technology at a large scale. On the other hand, biostimulation is the process of modifying environmental conditions such as substrates, nutrients, and electron acceptors to improve indigenous microorganisms with desirable metabolic capabilities^35^. Thus, the potential of biostimulation is required to be explored in detail for soil strength improvement.

Even after such a vast detailed study of the MICP in geotechnical engineering, a very limited study has been observed to improve the engineering properties of the clayey soil using MICP^36^. The major challenge to implement the MICP in clayed soil is its low permeability, which induced difficulty in the treatment process and therefore increased the cost of the ground improvement.

This study explored the effect of indigenous bacteria present in the soil to stabilized swelling behavior and improved the mechanical property of expansive soil. The objective of the research is to investigate the effectiveness of the biostimulation microbial induced calcite precipitation (MICP) for controlling the swelling-shrinkage behavior and improving the shear strength of expansive soil. An attempt was made to develop an effective procedure to culture the indigenous bacteria for treating clays with varying plasticity and improve their engineering behavior.

## Background

MICP is the multidisciplinary, well-recognized, potentially sound, environmentally friendly, and sustainable method for strength enhancement of soil. Sharma et al.^37^ presented the effective used of Bacillus sphaericus and Sporosarcina pasteurii along with algae to immobilize the lead contamination and increase the engineering property of Indian sandy soil^37^. Effective use of MICP technique from lab scale to field scale has been demonstrated to improve the engineering characteristics of sand due to ease in treatment method^38–41^. Gomez et al.^39^ explore the potential of native bacteria to investigate the biocementation effect on sand. Past studies (Burbank et al. 2011; Burbank et al. 2013; Gomez et al. 2018; Tsesarsky et al. 2018) showed that biostimulation is a superior alternative because the bacteria are already accustomed to the soil environment compared with augmented bacteria. However, the method is still underexplored for strength improvement of clayey soils. Only a few studies have been carried out to investigate the biostimulation and mechanical stabilization of clays.

The effective pore diameter in clayed soil to accommodate the bacterial strain has been studied by Ref.^42^ and observed that the pore size in 1.5 times larger than the bacterial strains. Li et al. studied the effect of bacillus megaterium along with fly ash at micro and macro levels to improve the engineering characteristics of the expansive soil. The study shows that biocementation with fly ash treatment improved the long-term performance of the expansive soil^43^. Sikha studied the suitability of MICP method to improve the expansive soil and investigated the effect by conducting Atterberg's consistency limit, unconfined compressive strength (UCS), and consolidation tests^44^. The increase in the UCS strength and reduction in the swelling behavior were reported in the study. However, minimal change in Atterberg's limit has been observed for highly plastic clay. To overcome this Cardoso et.al. application of MICP to sand-kaolin mixture and investigated the pore-clogging and compressibility effect due to biocementation^45^. Liu et al. explored the effect of bioremediation technique to reduce the desiccation cracking in the clay soil using bacterial and cementation solution and verified the applicability of MICP to improve the crack resistance capacity of clayey soil under wetting–drying conditions^46^.

Chittoori et al. explored the effectiveness of native bacteria to indue the biocementation in the expansive soil since most soil bacteria could precipitate Calcite. The effectiveness of the native bacteria to precipitate the calcium carbonate has been verified by monitoring the swelling behavior and unconfined compressive strength. The results show the effectiveness of the treatment method for the low plasticity clay^47^. For simulating the native bacteria in the clayey soil, substrate solution needs to flow in soil. However, due to the low permeability of clayey soil, it is highly difficult to percolate the flush solution under gravity; therefore, the flushing of the solution needs to be carried out under high pressure. However, the study carried out by Chittoori and Neupane, shows that the application of high pressure for the road subgrade improvement may generate cracks in the soil^27^. Therefore, Chittorri developed an alternative mixing method of substrate solution similar to the fly ash, cement treatment protocol^48^.

## Materials and methods

### Soil types

The three-soil type with varying plasticity index has been considered in this study. The soil has been collected from Madhya Pradesh (India). The biostimulated MICP treatment has been carried out on three natural soil along with the three artificial soil. The artificial soil has been prepared by mixing the 30% bottom ash in expansive soil to reduce the plasticity index. The expansive soil was modified by adding the dry mix of 30% BA. Sudhakaran et al. investigated the effect of bottom ash on clayey soil engineering properties and found that 20% and 30% BA give an almost similar improvement in UCS and can be considered as optimum limit^49^. Therefore, the optimum proportion proposed by Sudhakarn et al. has been taken to achieve the maximum effect of BA along with the MICP treatment. Here the natural soil is referred to as BC-1, BC-2, and BC-3, and artificial soil has been referred to as BC-1 + 30%BA, as BC-2 + 30%BA, and as BC-3 + 30%BA. As a result, the effect of MICP has been investigated on six soil types. The various index properties of the natural and artificial soil have been investigated as per Indian Standard. The obtained results are tabulated in Table 1.

**Table 1:** Index properties of considered soil.

Property	Soil type					
	BC-1	BC-2	BC-3	BC-1 + 30%BA	BC-2 + 30%BA	BC-3 + 30%BA
Liquid limit (%)	87	69.67	63.51	65.25	51.55	45.09
Plastic limit (%)	43	32.64	35.37	39.73	27.89	22.77
Plasticity index (%)	44	37.03	28.14	25.52	23.66	22.32
Clay (%)	71.5	63.50	57.20	50.05	44.45	40.04
Silt (%)	24.5	31.7	37.4	44.75	49.31	52.94
Sand (%)	4.0	4.80	5.40	5.2	6.24	7.02
Free swell index (%)	120	85	60	65	40	25
Optimum moisture content (%)	19.2	18.62	17.93	18.03	17.25	16.65
Maximum dry unit weight (kN/m^3^)	17.65	17.79	18.02	18.54	18.25	18.65

### Treatment procedure

In order to achieve uniform calcite precipitation in the treated expanse of soil, biomineralization has been performed by providing the enrichment and cementation solutions. The most specific task was to provide a suitable environment to grow the indigenous bacteria in the expansive soil. The enrichment solutions are used to increase the culturing of the gram-positive bacteria and, therefore, to provide a conducive eco-system for bacterial growth; the enrichment solution is mixed with nitrogen, carbon, and other necessary nutrients. The enrichment solutions are prepared with 100 mM sodium acetate (C_2_H_3_NaO_2_), 333 mM urea (CH_4_N_2_O), and 2.0 g/L nutrient broth. The nutrient broth is a potent source of suitable minerals, vitamins, and amino acids, providing a supportive environment for native soil bacteria culture. The nutrient broth has been added in both cementing and enrichment solutions. The cementing solution is prepared to facilitate the eco-system that supports calcite precipitation. To prepare the cementing solution, 250 mM calcium chloride (CaCl_2_) was added to the enrichment solution. The presence of calcium chloride increases the rate at which calcium carbonate is precipitated. Both the solutions have been prepared with tap water to ensure the applicability of field conditions. The calcite content yielding capacity of cultured bacteria in cementation solution has been quantified. It has been observed that 1.28 g CaCO_3_ per mL solution was formed.

### Mixing protocol and sample preparation

The mixing protocol is the most challenging part of the MICP treatment of the expansive soil. The enrichment and cementation solution has been directly added to soils by considering the low permeable nature of expansive soil. The air-dried sample of the expansive soil BC-1, BC-2 and BC-3 were kept in an environmental chamber at 27 ± 2 °C temperature and 65 ± 5% humidity for 24 h before treatment. The soil is not oven-dried to preserve the bacterial presence. The optimum moisture content (OMC) has a well influential role in soil stabilization; therefore, initially, the enrichment solution has been added in the soil up to OMC of the respective soil type (Table 1). Thereafter to hydrolyze urea, the prepared soil mix has been again kept in an environmental chamber at 27 ± 2 °C temperature and 65 ± 5% humidity for a different period of time (1, 2, 3, and 4 days). This period is known as the mellowing period and is referred to as MP-1, MP-2, MP-3, and MP-4 for 1, 2, 3, and 4 days. The loss of enrichment solution content has been determined after each mellowing period, and after that cementation solution was added to replace the loss percentage of the enrichment solution. The enrichment and cementation solution was directly mixed in the soil sample using the planetary mixture to ensure homogeneous mixing.

The cylindrical specimens of 38 ± 2 mm diameter 76 ± 4 mm length were prepared to examine the confined compressive strength treated and untreated soil. The sample was again placed in the environmental chamber at 27 ± 2 °C temperature and 65 ± 5% humidity to maintain the constant temperature in the compacted samples. To perform the 1-D swell test, the soil has been compacted, and then the core of size 60 mm diameter and 20 mm height has been extracted from each soil mix. For the microstructural assessment, the soil samples have been taken from UCS samples after testing.

### Test carried out

**Figure 1:**
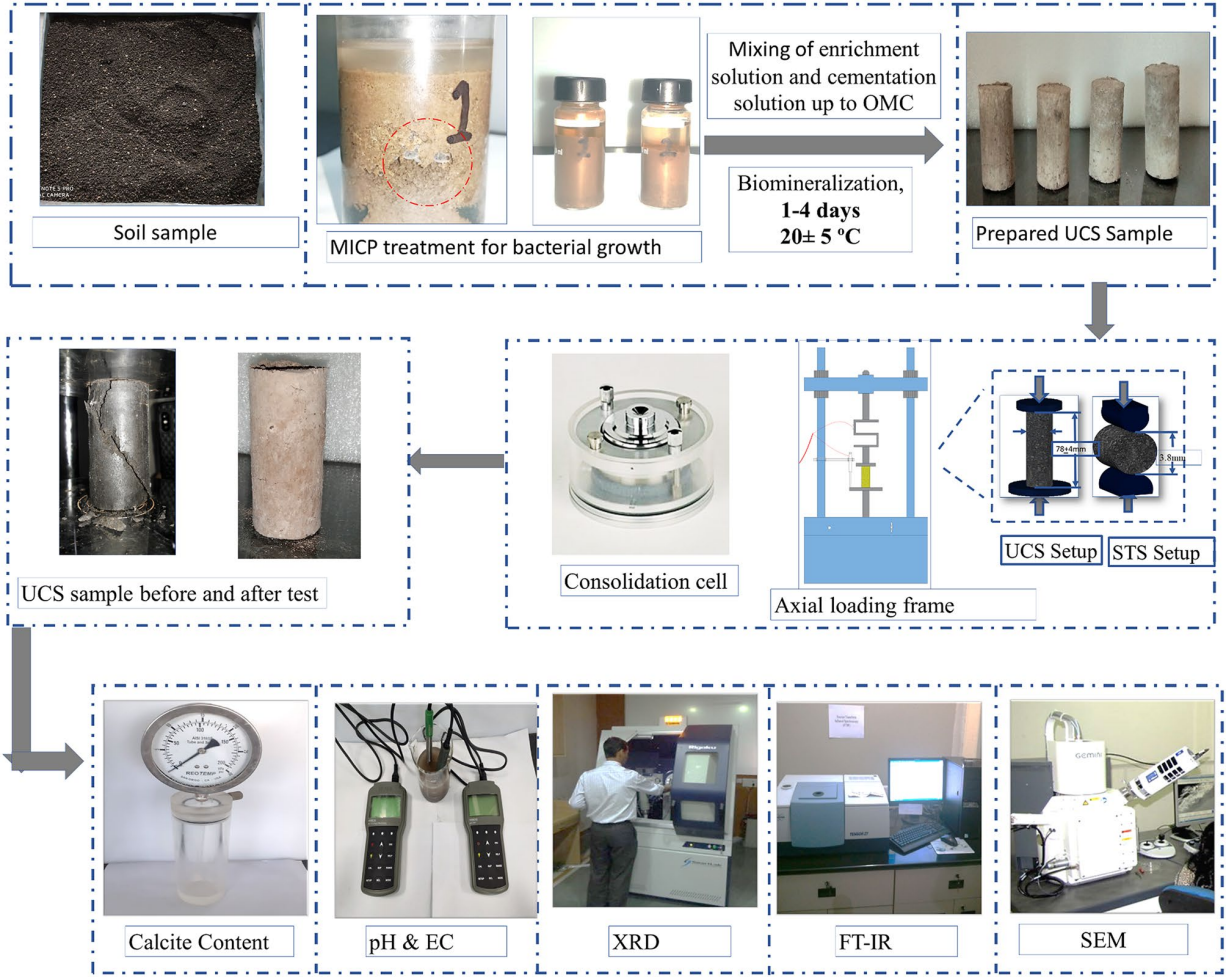
A detailed procedure of bacteria culturing and test setup.

The effect of indigenous bacteria to precipitated the calcite content and stabilized the expansive soil for the road pavement has been assessed through various chemical, microstructural, mechanical tests. The shear and tensile strength of the treated and untreated specimens was assessed by carrying out the unconfined compressive strength (UCS), and split tensile strength (STS) tests. The UCS test was carried out as per Indian Standard IS 2720 (Part 10):1991. The specimen for each sample combination were prepared by maintaining the length and diameter ratio 2. The UCS test was conducted on a fully automatic mechanical load frame. The vertical displacements and deformation load were recorded using LVDT of 25 mm and an S-type load cell of 2.5 kN capacity. The strain rate of 1.25 mm/min was applied to observed the stress–strain behavior of treated and untreated expansive soil. The split tensile strength test was conducted according to the Brazilian split tensile strength (STS) test procedure as per IS 10082:1981. The load was applied on lateral dimension at a 1.25 mm/min strain rate during STS testing. The compacted specimen was curred in a moisture chamber at 20 ± 2 °C temperature and 100 ± 5% humidity for 7 days. The swell pressure and swell strain were examined using a 1D swell test as per IS 2720 (Part 15); 1986. The specimen of size 60 mm diameter and 20 mm thickness were prepared at a liquid limit to carry out the 1D Swell test. The liquid limit, plastic limit, and free swell index of each mellowing period of treated soil were determined as per IS 2720 (Part 5):1985 and IS 2720 (Part 40):1977, respectively. The three replicas of each sample combination were prepared to understand the test results' reproducibility for complete testing. After each mellowing period, the stabilization of expansive soil resulted in the change in soil structure's chemical and microstructural arrangements. The quantification of the chemical alternation in expansive soil after different mellowing periods has been investigated by carrying out pH, electrical conductivity, and calcite content tests. The calcite content formation is the direct reflection of the effectiveness of the treatment procedure. The electrical conductivity and pH signify the change in the chemical state of the expansive soil. The mineralogical characterization of powder samples using XRD has been carried out on all the treated specimens. Similarly, to understand the change in the chemical bond during the treatment process, ATR-FTIR analysis has been carried out on KBr pellet specimens. The IR scans have been made from 400 cm^−1^ to 4000 cm^−1^ using the ATR-FTIR spectrometer (PerkinElmer). The microstructural assessment of soil matrix has been studied using scanning electron microscopy (SEM) micrograph). The freeze-cut-dying method has been used to prepare the sample for SEM since this method preserves the original microstructure^50^. The complete test procedure has been shown in Fig. 1.

## Results and discussion

The indigenous bacteria's effectiveness in stabilizing the expansive soil has been extensively examined through several engineering test procedures to apply the method for road pavement. This section presentation test results with appropriate reasoning of the alternation in the state of the soil matrix. The results reported in this section are taken from an average of three identical specimens with similar experimental conditions.

### Effectiveness of the treatment on plasticity and swelling characteristics

**Figure 2:**
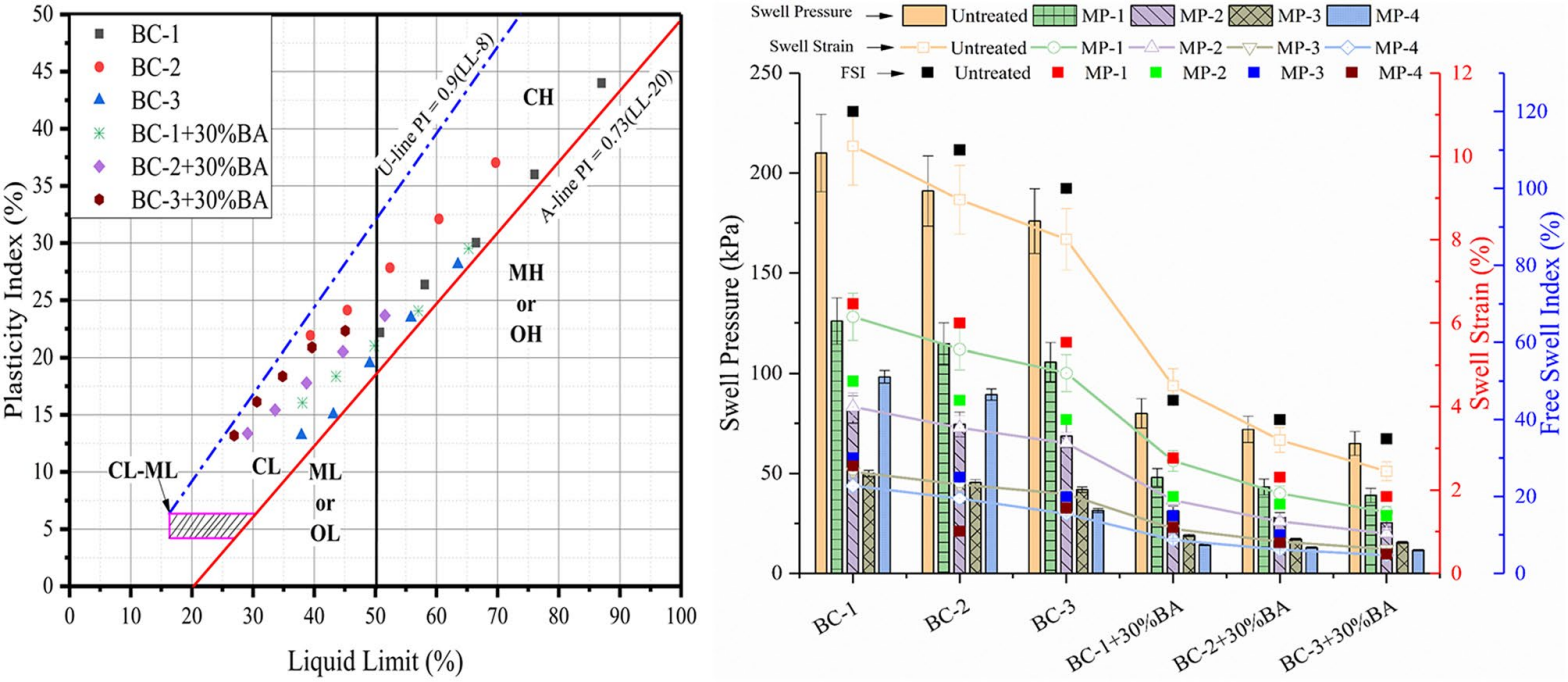
Effect of biostimulated MICP treatment on plasticity and swell properties of the expansive soil.

The liquid limit and plastic limit of the treated and expansive untreated soil have been investigated to assess the treatment procedure's effectiveness. After treatment, the biotreated expansive soil specimen has been crushed and oven-dried at 105 ± 5 °C temperature before investigating Atterberg's limit. The effectiveness of Bio stimulated treatment of the expansive soil on plasticity characteristics is shown in Fig. 2. The Atterberg's limits have been assessed on natural and artificial soil with different mellowing periods. It has been observed that initially, the soil was classified as highly plastic clay (CH) in accordance with a unified soil classification system. But after the treatment protocol, it turned into low plasticity clay (CL). The reduction of liquid limit and plasticity with the addition to the cementation solution and the increase of the mellowing periods can be attributed to the microbial activities. As the mellowing period increases, microbial activities increase, and more bacteria are formed in longer exposure to the aerobic environment, which initiate MICP activity, and as a result, the slime precipitates. The bacterium process supports the clogging of the soil particles and also initiates the cementation effect^51^. The cementation mineral formed in the soil improves the adhesion of soil particles and increases the cohesion, which reduces the liquid limit of the expansive soil. The reduction in plasticity behavior was also found due to the formation of calcite in the pores of the soil matrix, which increases the binding of soil particles^52^. It is also observed that artificial soil has a high reduction due to the bottom ash pozzolanic reaction.

The free swelling index for the natural and artificial soil has been calculated to understand the treatment's initial effect on swell potential. The impact of different mellowing periods and test protocol on a free swell index is present in Fig. 2. For the natural soil BC-1, BC-2 and BC-3, the FSI have been observed as 120%, 110%, and 100%, respectively. And for the artificial soil BC-1 + 30%BA, BC-2 + 30%BA and BC-3 + 30%BA, the FSI has been reduced to 45%, 40%, and 35%, respectively. After mellowing period MP -4, the FSI is limited to 15–30% for natural soil and 5–15% for the artificial soil. This exponential reduction in the FSI indicates the enrichment solution and cementation solution effectively provide an eco-system to culture the urease producing indigenous bacteria to precipitate the calcium carbonate. The calcite mineral formed due to the precipitation of calcium carbonate is non-expansive and induced a cementation effect between soil particles. In SEM micrograph, it was observed that during the MICP treatment, a layer of calcite content forms on the soil particles. Thus, the specific surface of expansive hydrophobic clay minerals reduced and the content of calcite formed on the soil particles prevents the reaction of soil and water particles. As a result, a reduction in FSI was observed. It can also be seen as more bacteria are formed in an aerobic environment in longer mellowing period conditions, and therefore a higher reduction in FSI was observed.

Further to assess the swelling pressure and swell strain, a 1-D swell test has been conducted, and results are shown in Fig. 2. For the natural soil BC-1, BC-2, and BC-3, the swell pressure has been observed as 210 kPa, 191 kPa, and 176 kPa, respectively. The exponential reduction in the swell pressure has been observed with MICP treatment. The swell pressure has been reduced up to 41 kPa for natural soil and 15 kPa for artificial soil. The MICP treatment procures the calcite content, which creates the bond between the soil grains and the formation of the biofilm also increased the barrier between water and clay particles^53,54^. And for the artificial soil BC-1 + 30%BA, BC-2 + 30%BA and BC-3 + 30%BA, the swell pressure has been reduced to 48 kPa, 43 kPa, and 39 kPa %, respectively. The reduction in swelling pressure is attributable to the pozzolanic reaction of the bottom ash content. The possible reason for the reduction in swelling pressure can also be observed due to the addition of silt-sized bottom ash particles in the expansive soil. Bottom ash provides a polyvalent cation that induces flocculation of soil particles^55^. The specific surface of the expansive clay mineral was reduced due to the addition of the silt-sized particles of the bottom ash and the formation of the C–S–H gel during a pozzolanic reaction. As a result, a reduction in swelling pressure was observed. A similar trend has been observed for the swell strain, as shown in Fig. 5. For the natural soil BC-1, BC-2 and BC-3, the swell strain has been observed as 10.25%, 8.96%, and 8.01%, respectively. And for the artificial soil BC-1 + 30%BA, BC-2 + 30%BA and BC-3 + 30%BA, the swell strain has been reduced to 4.5%, 3.2%, and 2.45%, respectively. The swell strain has been reduced to 1.4% for the BC-3 soil after MP-4 and for the artificial soil it has been reduced to up to 0.43%. These values lie under the acceptable limit of 1.5% for the swell strain. However, the swell strain for BC-1 and BC-2 has been observed as 2.43% and 2.13%, which is bit higher than the acceptable limit of 1.5%. The results depict that for high plastic clay, the treatment period may be required to increase. The MICP treatment effectively reducing the swell pressure and swellstrain, which shows that the biostimulated treatment of the expansive soil can effectively control the swelling behaviors of the clayey soil.

### Effectiveness of the treatment on chemical bonds, mineralogy, and microstructure

**Figure 3:**
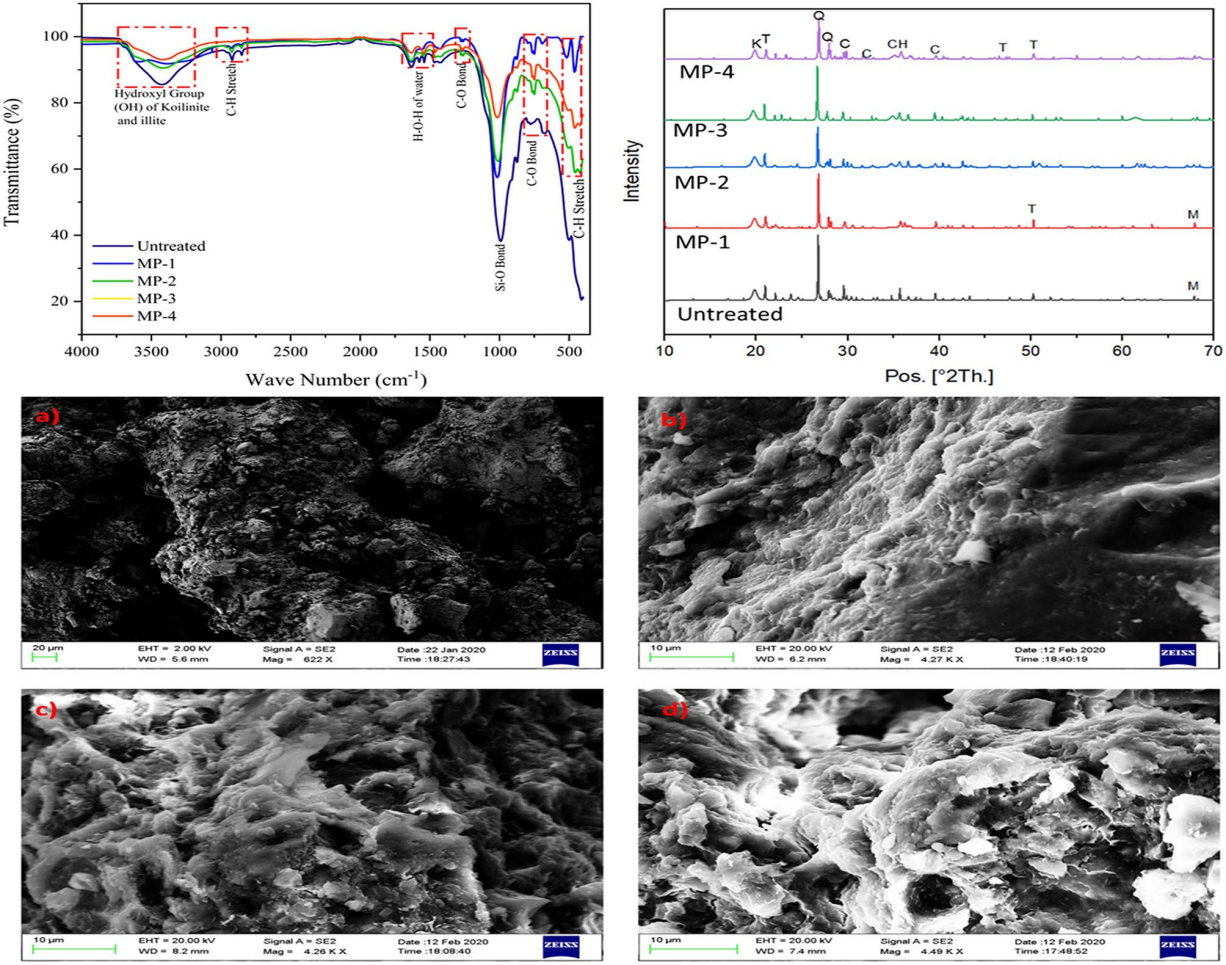
FTIR, XRD and SEM micrograph of MICP untreated and treated expansive soil.

The IR analysis has been performed on the varying mellowing periods of MICP treatment and untreated BC-3 soil (Fig. 3). The IR spectra of each mellowing period have been compared with for BC-3 soil. The IR range of 500–1200 cm^−1^ shows the mineral, 1200–3000 cm^−1^ organic matters and 3500–4000 cm^−1^ clay minerals^21^. The hydroxyl group of kaolinite and illite has been observed in the IR band of 3092–3717 cm^−1^. The presence of the moisture content is observed at 3433 cm^−1^. A broad IR peak has been observed at the 3454 cm^−1^ that shows the presence of calcium carbonate formation in the treated specimen^56^. An intensified peak has been observed with MP-4, which shows a higher amount of calcite formation. A C–O bond peak has been observed at 874 cm^−1^ and 1468 cm^−1^. The increasing mellowing period reduced the C–O bond peak, which ascertained the reduction in the carbonate^57^. The division in carbonation peak was obtained at 1468–1430 cm^−1^ due to the partial carbonation of the hydration product^58^. The presence of the quartz was obtained at the IR peak of 798 cm^−1^ and 782 cm^−1^. The IR peak was observed at 3623 cm^−1^ ascertained the presence of montmorillonite mineral in the untreated BC soil. This peak has been reduced by increasing the MICP treatment cycle. This shows the MICP treatment controls the presence of hydrophobic minerals.

The mineralogical changes due to varying mellowing periods of MICP treatment in the expansive soil have been analyzed using X-ray diffraction (Fig. 3). The formation of the calcite content and portlandite has been observed in XRD pattern. In all the specimens, quartz has a highly distinct peak; however, the intensity has been reduced with an increased mellowing period. It is evident that due to the formation of calcium carbonate, the presence of the Tobermorite mineral was observed. The XRD pattern shows the sharp peak of the tobermorite mineral in MP-4 sample, which shows the higher calcite content is produced in this mellowing period for 4 days.

The scanning electronic microscopy analysis has been carried out to analysis the effect of MICP treatment on expansive soil (Fig. 3). The SEM micrograph of untreated soil, MP-2, MP-3, and MP-4 mellowing periods are present in Fig. 3. It can be observed in Fig. 3 that there are various cavities in the untreated soil. These cavities have been filled with calcite precipitation as observed in SEM micrographs). It can also be seen that with an increase in the mellowing period, the quantity of the calcite content increased. A similar trend has been observed in calcite content analysis. The SEM analysis ascertains the effectiveness of the method to culture the indigenous soil bacteria for calcite precipitation.

### Effectiveness of the treatment on pH, CCt, EC

**Figure 4:**
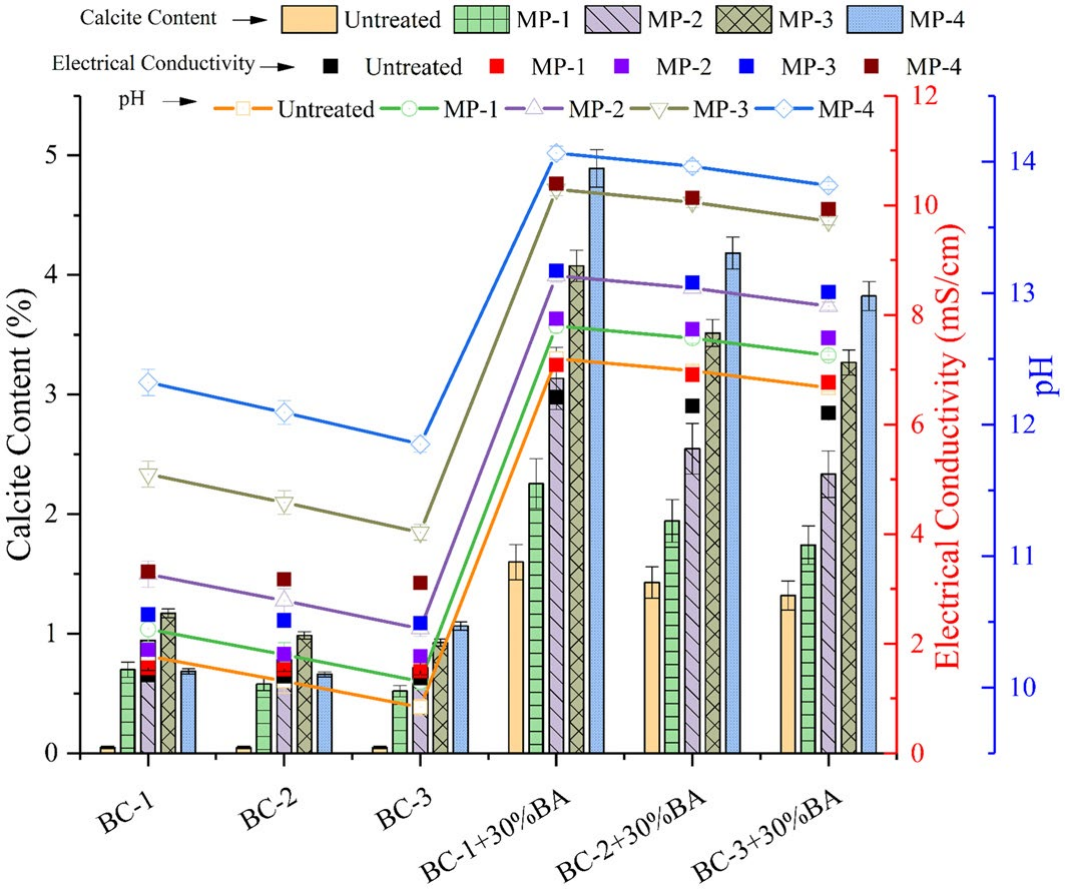
Effect of biostimulated MICP treatment on chemical properties of the expansive soil.

The chemical alternation with the varying mellowing periods of MICP treatment are presented in Fig. 4. The value of the electrical conductivity (EC) is increased with the increasing mellowing period. The increase in the EC values depicts the chemical alternation in the soil matrix. The increase is due to the conversion of non-ionic compounds into ionic compounds. The increase in ionic concentration was due to urea hydrolysis during biogeochemical reactions. The increase in EC was found to be greater during the cementation cycle than during the stimulation period, as calcium chloride dihydrate was additionally mixed during the treatment cycles. When the calcium source was added in chemical solution, different anions produced complexes dissolved with calcium. The complexes include calcium bicarbonate and calcite hydroxide. The greater the number of ions generated during the cementation cycles, the more increase in EC observed. The increase in EC shows the successful execution and completion of biogeochemical reactions. For the natural soil BC-1, BC-2, and BC-3, the EC has been observed as 1.43 mS/cm, 1.40 mS/cm, and 1.37 mS/cm, respectively. And for the artificial soil BC-1 + 30%BA, BC-2 + 30%BA and BC-3 + 30%BA, the EC has been increased to 6.5 mS/cm, 6.34 mS/cm, and 6.21 mS/cm, respectively. After mellowing period MP-4, the EC is increased to 3.11–3.31 mS/cm for natural soil and 9.29–10.39 mS/cm for the artificial soil.

The pH of the MICP treated soil was raised, and it depicts the change in the nature of the mixture toward acidic nature. Goodarzi et al. show that the increase in the pH values also ascertained the increment in shear strength of soil^59^. For the natural soil BC-1, BC-2, and BC-3, the pH has been observed as 10.24, 10.05, and 9.85, respectively. And for the artificial soil BC-1 + 30%BA, BC-2 + 30%BA and BC-3 + 30%BA, the pH has been increased to 12.5, 14.41, and 12.28, respectively. After mellowing period MP-4, the pH is increased to 11.85–12.32 for natural soil and 13.81–14.06 for artificial soil.

The calcite content test has been conducted to quantify the percentage of the Calcite in MICP treated specimens. For the artificial soil BC-1 + 30%BA, BC-2 + 30%BA and BC-3 + 30%BA, the CCt has been increased to 1.6%, 1.43%, and 1.32%, respectively, but for the natural soil, no calcite content has been observed without treatment. After mellowing period MP-4, the CCt is increased to 0.91–1.17 for natural soil and 3.82–4.89 for artificial soil. This signifies that the higher concentration of the MICP treatment formed more calcite in the specimen. Higher calcite content formation improves soil matrix and, as a result, improves the strength and durability of the stabilized expansive soil. For the natural soil, calcite content has been increased up to 134% and 205% for artificial soil. The increase in the calcite content ascertained the effective treatment of both natural and artificial soil with culturing the indigenous bacteria.

### Effectiveness of the treatment on strength characteristics

**Figure 5:**
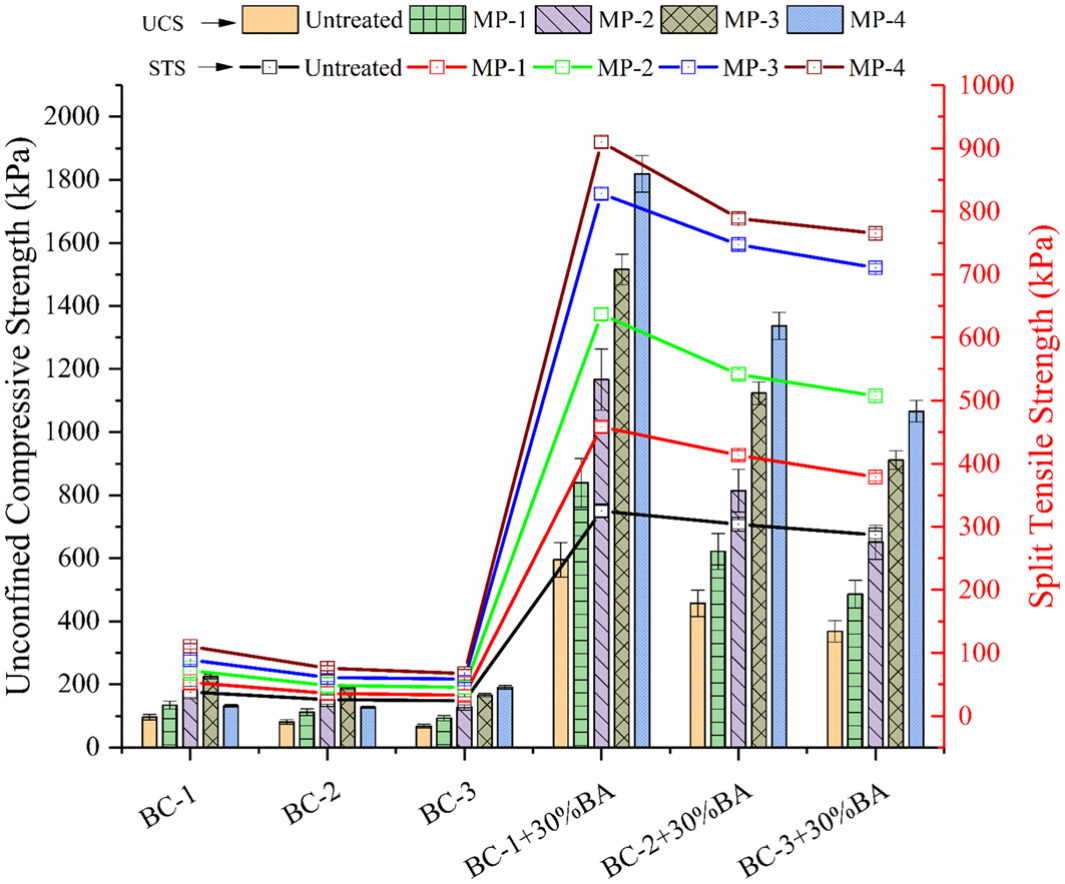
Effect of biostimulated MICP treatment on unconfined compressive strength and split tensile strength of the expansive soil.

The unconfined compressive strength of the expansive soil with a varying mellowing period for both natural and artificial soil with MICP treatment, as shown in Fig. 5. The effect of four mellowing periods on six different soil with and without treatment is investigated. The UCS values have been calculated to understand the undrained shear behavior of the expansive soil. An increase in strength with the addition of MICP treatment can be ascertained due to the effectiveness of the enrichment and cementation solution to culture the indigenous soil bacteria for calcite precipitation. For the natural soil BC-1, BC-2 and BC-3, the UCS has been observed as 96 kPa, 81 kPa, and 68 kPa, respectively. And for the artificial soil BC-1 + 30%BA, BC-2 + 30%BA and BC-3 + 30%BA, the UCS has been increased to 595 kPa, 437 kPa, and 368 kPa, respectively. After mellowing period MP-4, the UCS is increased to 165–225 kPa for natural soil and 1066–1819 kPa for the artificial soil. The UCS value has been increased by 134% for natural soil and 210% for artificial soil. Although the values of UCS have been increased for the natural soil up to 134%, these values are still below the threshold (1.75 MPa) value required for the road pavement subgrade as per MoRTH (2013)^60^.

The increase in the UCS value has been observed with MICP treatment; however, it is also increasing the brittle nature of the treated specimen. The split tensile strength test has been carried out to understand the effect of the MICP method on the tensile strength. The Tensile strength considerably impacts the shrinkage behavior of the expansive soil. It has also been carried out to understand the brittle behavior of the treated expansive soil. All the treated specimens have been tested for the split tensile strength to explore the effect on tensile cracking mitigation in expansive soil (Fig. 5). The calcite content precipitation increases the interfacial interaction between the soil particles, which increases the tensile strength in the expansive soil. For the natural soil BC-1, BC-2 and BC-3 the STS have been observed as 38 kPa, 26 kPa and 24 kPa, respectively. And for the artificial soil BC-1 + 30%BA, BC-2 + 30%BA and BC-3 + 30%BA, the STS has been increased to 325 kPa, 304 kPa and 287 kPa, respectively. After mellowing period MP -4, the STS is increased by 190% for natural soil and 180% for artificial soil.

### Statistical analysis

**Figure 6:**
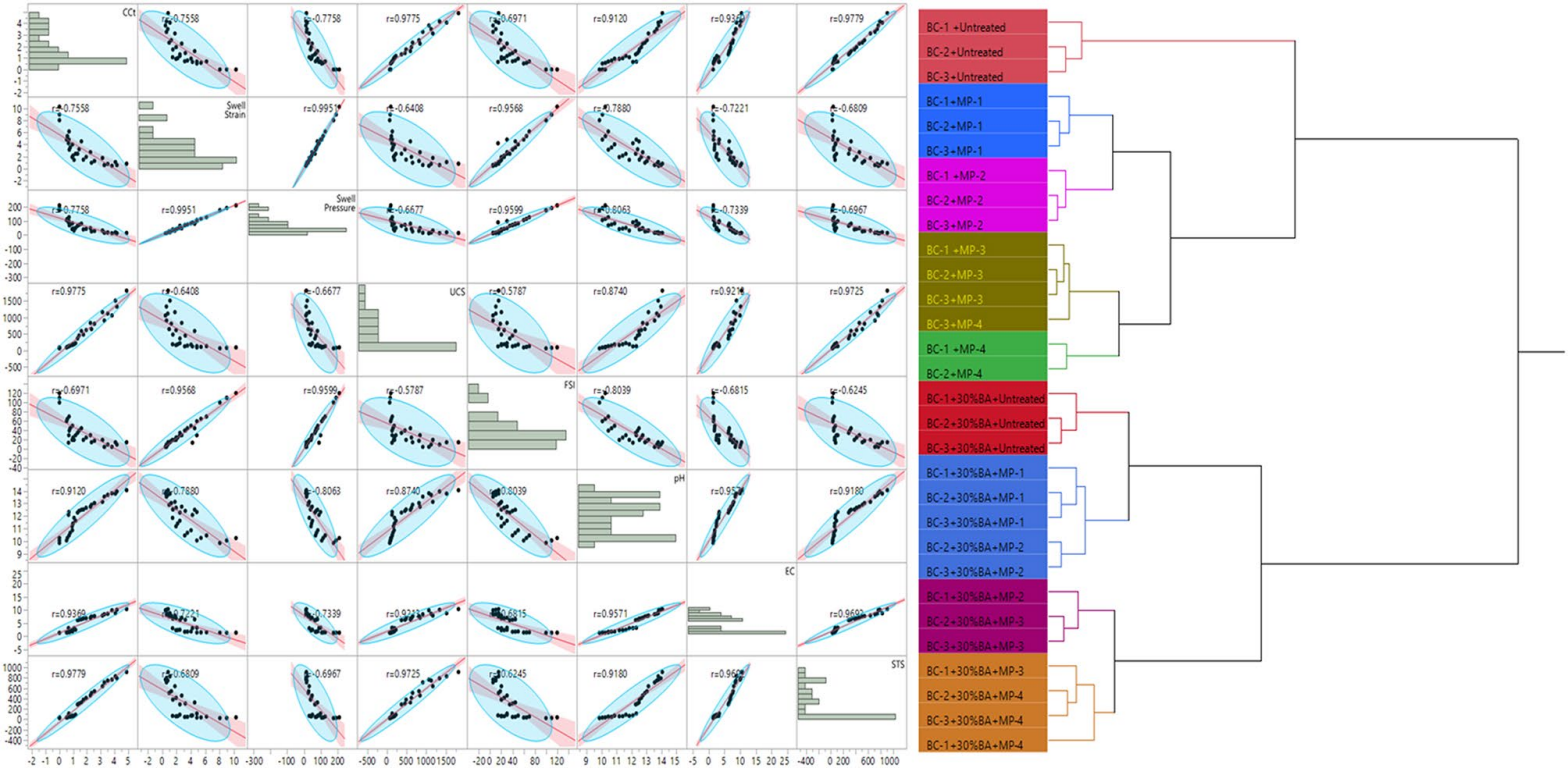
Multivariate scatter and hierarchical clustering dendrogram plot of MICP treated expansive soil.

To develop a significant relation among various mechanical and chemical analysis, multivariate analysis has been performed. The multivariate analysis has been carried out by considering different variables, i.e., pH, Calcite content, free swell index, swell pressure, swell strain, unconfined compressive strength, and split tensile strength. The relationship matrix has been developed as shown in Fig. 6. Here it is clearly observed that there is a close relationship between the swell strain and swell pressure; the value of R is observed 0.99. At the same time, if compare a result of swell pressure or free swell index with the UCS value, then R is observed as 0.57. The lower values show there is no close relationship between swell potential and UCS. The effect of calcite content on UCS and STS very close; here it can be concluded that with the increase in calcite content, the value of UCS and STS increased. And a similar trend has been observed with EC and pH. The Multivariate analysis clearly established the relationship between different parameters assessed in this study.

The hierarchical clustering of the complete experimental results has also been plotted as shown in Fig. 6. It can be observed from the presented dendrogram that the effect of the treatment period is more or less equal in every soil type. However, there is a slight change in the group with MP-4 on BC-3 soil, which is also showing similar results as shown by MP-3 treatment. In all, the treatment period has shown similar effects irrespective of the soil type.

### Limitation and future scope

This study was carried out to investigate the effect of the indigenous bacteria to improve the engineering properties of the expansive soil using MICP treatment. In the current study, laboratory-scale testing was carried out to understand the engineering behavior of biotreated expansive sol. However, to understand the actual behavior of MICP treated expansive soil, large-scale testing is required. The present study does not deal with the durability attributes and environmental implications of biotreated expansive soil; hence study on various environmental exposes can be conducted to assess the long-term performance. At the microstructure level, the identification of bacteria for the better cementation effect needs further assessment. The quantification of the biofilm formed during the treatment procedure is not investigated in this study; hence this can also be considered in the future scope of the work.

## Conclusions

This study is carried out to investigate the effect of indigenous bacteria to stabilized expansive soil subgrade using microbial induced calcite precipitation (MICP). The major challenge to implement the MICP in clayed soil is its low permeability, which induces difficulty in the treatment process and, therefore, increases the ground improvement cost. The alternative approach to direct mix the enrichment and cementation solution has been adopted to assess the viability of the biostimulated MICP treatment on clay for road pavement application. Three types of natural soil and three artificial soil (Natural soil + 30% bottom ash) has been considered to investigate the effect of MICP for 1–4 days mellowing period. Based on the detailed experimental and statistical investigation, the following conclusions have been drawn.

- The plasticity of the biostimulated MICP treated expansive soil has been deceased and soil classification has been changed from highly plastic clay (CH) to low plastic clay (CL). This alternation of the plasticity Index future reduces the free swell index, swelling pressure, and swell strain of treated soil. The swell pressure has been reduced up to 41 kPa for natural soil and 15.46 kPa for artificial soil.
- The unconfined compressive strength and split tensile strength of the biostimulated MICP treated expansive soil has been increasing with the increase in the calcite content, pH, and electrical conductivity.
- A considerable amount of calcite content has been observed with varying mellowing periods for the MICP treatment. The calcite content's presence confirms the effectiveness of the method used for culturing the indigenous bacteria to stabilize the expansive soil. The calcite precipitation filled the soil cavities and henceforth produced a strong soil matrix, as observed in the SEM micrograph.
- The statistical assessment using multivariate analysis and hierarchical clustering dendrogram shows the similar effect of the MICP treatment protocol on different soil. A very strong relationship between the calcite content and mechanical property is observed.
